# Cellular GFP Toxicity and Immunogenicity: Potential Confounders in in Vivo Cell Tracking Experiments

**DOI:** 10.1007/s12015-016-9670-8

**Published:** 2016-07-19

**Authors:** Amir Mehdi Ansari, A. Karim Ahmed, Aerielle E. Matsangos, Frank Lay, Louis J. Born, Guy Marti, John W. Harmon, Zhaoli Sun

**Affiliations:** 1 Department of Surgery and Hendrix Burn/Wound Laboratory, Baltimore, MD USA; 2 Department of Surgery, The Johns Hopkins University School of Medicine, Baltimore, MD USA

**Keywords:** Green fluorescent protein (GFP), Cytotoxicity, Immunogenicity, Reporter gene, *In vivo* cell tracking, Cell death

## Abstract

Green Fluorescent protein (GFP), used as a cellular tag, provides researchers with a valuable method of measuring gene expression and cell tracking. However, there is evidence to suggest that the immunogenicity and cytotoxicity of GFP potentially confounds the interpretation of *in vivo* experimental data. Studies have shown that GFP expression can deteriorate over time as GFP tagged cells are prone to death. Therefore, the cells that were originally marked with GFP do not survive and cannot be accurately traced over time. This review will present current evidence for the immunogenicity and cytotoxicity of GFP in *in vivo* studies by characterizing these responses.

## Introduction

Green fluorescent protein (GFP) was discovered in 1961 as a byproduct of the extraction of aequorin from the *Auquorea victoria* jellyfish [1]. The purification, crystallization [2] and subsequent description of the structural composition of the GFP chromophore [3] paved the path toward cloning of the GFP cDNA [4, 5]. The successful expression of GFP led to the widespread usage of GFP as a gene expression marker and a molecular and cellular tag.

**Fig. 1:**
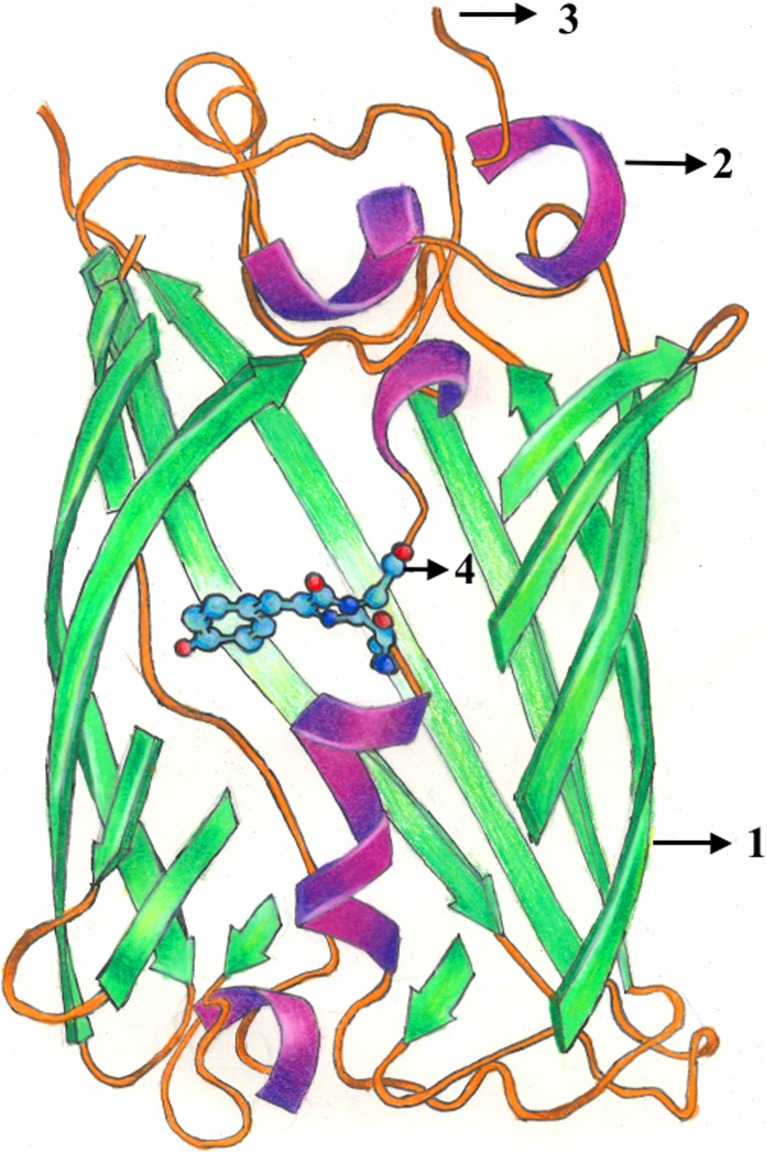
Molecular Structure of GFP. The tertiary structure of GFP is a beta barrel structure made up of 11 antiparallel β-strands (*1*) and 6 center-positioned alpha helices (*2*). Interrupting the stranded alpha helix, there are short helical loops on the ends of the cylindrical structure (*3*). A covalently bonded chromophore, 4-(p-hydroxybenzylidene) imidazolidin-5-one, is located and protected at the center of the structure (*4*)

GFP is composed of 238 amino acids (27 kDa), but only 4 amino acids directly produce fluorescent effects [6]. The folding and molecular structure (Fig. 1) both play integral roles in GFP’s fluorescent properties [7]. The formation of the functional chromophore in GFP, and that of analog proteins, entails three essential steps: protein folding, cyclization of the tripeptide chromophore motif, and oxidation of the cyclized chromophore [8–10]. The chromophore is responsible for the absorption of ultraviolet radiation. Upon excitation, proton transfer allows energy to be released in the form of visible photon emission. Wild-type GFP has a major excitation peak around 395 nm, a minor excitation around 475 nm, and a primary emission peak around 509 nm [11–13].

GFP has been engineered to produce an assortment of useful mutants, such as red, cyan, and yellow fluorescent proteins. Additionally, other fluorescent proteins, ranging from orange to the far-red spectral regions, have been manufactured [14–17]. When assessing fluorescent proteins for a particular experimental use, it is crucial to consider many features including: photo/temperature-stability as well as color intensity, cytotoxicity, and immunogenicity [18]. For most purposes, binding of GFP to a cell surface epitope is the important mechanism by which GFP labels cells. Fluorescent protein fusion is another method that targets proteins and tags their expression. This eliminates the need to label, purify, or deliver the protein into the cell. This makes it possible to label and observe a protein’s dynamics, history, steady state distribution, and association with other proteins in what were previously inaccessible cell environments [19, 20]. However, fluorescent protein (FP) fusions to native proteins are not small alterations. They constitute significant additions, which may have steric consequences for protein function, targeting, and folding.

GFP is widely utilized to label cells for tracking. However, the cellular damage, caused by GFP transfection/transduction may lead to misinterpretation of experimental results. The cellular damage is described as possibly resulting from direct injury by reactive oxygen species (ROS) generation, initiation of apoptosis, and damage by immune mechanisms. [10, 13, 21–24]. In this review, we will focus on the mechanisms of cellular damage caused by GFP-transfection as explored in various animal models.

## Immunogenicity of GFP (Table 1)

Extracellular antigens are taken up and associated with major histocompatibility complex (MHC) class II molecules in the endoplasmic reticulum (ER) via invariant chain (li) trafficking. However, MHC class I expression is applied for intracellular antigens, such as that of a viral infection, through TAP1/2 transport into the ER. GFP is most commonly utilized as a reporter gene insert. As such, it is processed in the host cell. It is postulated that GFP immunogenicity acts through T- cell mediated immunity [8, 11, 25, 26]. The immune response is elicited at the cell’s surface through class 1 MHC presentation and cytotoxic T-lymphocyte recognition of the GFP antigen [11]. The murine MHC class I, known as H-2 K/D/L, consists of extracellular α heavy chains primarily present on chromosome 17, and an α3-associated β2-microglobulin on chromosome 2 [27].

**Table 1:** Immunogenicity and cytotoxicity of GFP

GFP Immunogenicity	GFP Cytotoxicity
T-cell/MHC I medicated immunogenicity [11]	General photocytotoxicity [24]
Variation of T-cell mediated immunogenicity in different strains [6]	Induction of apoptosis [13]
Variation of T-cell mediated immunogenicity by rout of administration [6]	Oxidative stress [35, 36]
Immunogenicity in transplanted hepatocytes [7, 28]	Cytotoxicity of GFP and its analogs [21]
Diminished immunogenicity by immunosuppressant [8]	Cardiac cytotoxicity [38]
Extensive CD8+ T-cell reaction to GFP-induced CD4+ hematopoietic stem cells [26]	Actin-myosin dysfunction in muscle cells [39]
	Neurodegeneration [41]

Although immunogenicity of GFP and its variants share a similar mechanism through MHC class 1, animal model and route of in vivo administration can affect eGFP immunogenicity [6, 11]. To investigate the eGFP elicited cytotoxic immune response researchers utilized Balb/c mice and BM185 (transplantable murine model, pre-B leukemia) cell line. Typically, intravenously injected wild-type pre-leukemia cells cause systemic leukemia and mortality of the immunocompetent Balb/c mice; however, when transduced to express eGFP, the leukemia cells did not cause recipients deaths. In contrast to the response of the immunocompetent mice, intravenous administration of wild-type leukemia cells or eGFP-expressing leukemia cells to Nu/Nu mice, which lack functional T-cells and cytotoxic T lymphocyte (CTL) response, caused recipient mortality. Subsequent quantification of CTL response, in immunocompetent mice, demonstrated a three-fold increase in the CTL response against eGFP-expressing leukemia cells compared to non eGFP-expressing leukemia cells. This finding highlights the importance of T-cells in developing immunologic response against eGFP-expressing leukemia cells [11]. Interestingly, when pre-leukemic cells were subcutaneously administered to immunocompetent mice, all of the mice developed rapidly growing subcutaneous tumors in normal and eGFP-transduced cells. This suggested that the route of administration plays an important role in initiating an immune reaction against eGFP-expressing malignant cells [6].

To confirm and test the aforementioned findings in different mice strains, a similar experiment was carried out with C57BL/6 mice. Subcutaneous injections of EL-4 lymphoma cells (transplantable murine model, T cell lymphoma) to C57BL/6 mice caused palpable tumors 13 days after injections. However, eGFP-expressing EL-4 cells did not cause tumor growth in any of the recipients [11]. Confirming the importance of the route of administration in development of immunologic response against eGFP, transplantable T-cell lymphoma cells, administered intravenously, did not cause tumor growth in C57BL/6 mice regardless of whether or not the lymphoma cells were transduce with eGFP [6].

These findings suggest that eGFP is immunogenic in Balb/c mice whereas it is only slightly immunogenic in C57BL/6 mice. Both strain and route of administration play respective roles in the immune response of eGFP-transduced cells administered in vivo [6]. Consistent with previous experiments in mice, infusion of autologous eGFP-transduced CD34+ bone marrow derived hematopoietic stem cells to rhesus macaques led to lysis of the CD34+ cells and induced significant CD8+ mediated T-cell reaction [26].

There is also evidence to suggest that there is a rejection of GFP-expressing cells following transplantation to non-GFP-expressing wild-type animal, the effect then is diminished following immunosuppressant therapy. GFP expression in hepatocytes following transplantation is observably diminished over time. Hepatocytes obtained from GFP transgenic rats were transplanted into the livers of wild-type rats. For comparison, wild-type hepatocytes were transplanted into GFP-transgenic rat livers as well [7]. After showing engraftment using fluorescent microscopy, the transplanted hepatocytes were tracked for 48 days. Implying an underlying damaging process, GFP-positive hepatocytes in wild-type rats livers decreased more rapidly than the wild-type hepatocytes transplanted into the GFP transgenic rat livers. Moreover, GFP-positive hepatocytes attracted CD4+ and CD8+ infiltrating inflammatory cells, which is consistent with an immunological response [7]. Additionally, immunologic modification by bone marrow transplantation and administration of Tacrolimus, a T-cell inhibitor agent, showed increased survival of transplanted GFP-positive hepatocytes. Host pre-immunization with GFP-positive hepatocytes led to complete loss of GFP-positive hepatocytes by day 14 [7].

The results of this study are also congruent with the results of a GFP gene transfer study into the liver of mice where, GFP-induced hepatocytes decreased or disappeared in immunocompetent livers 2 weeks after transplantation [28, 29].

To further explore the immunogenicity of the GFP protein, the effectiveness of the immunosuppressant Cyclosporine was studied in preventing an immune response to GFP in dogs transplanted with GFP-transduced CD34^+^ hematopoietic stem cells [8] Low GFP expression was associated with more potent T-cell immune responses to the GFP, when compared to non-transplanted controls. Dogs treated with cyclosporine after hematopoietic stem cell transplantation showed stable GFP expression for over 800 days. This suggests that immunosuppression prevents immunoactivation against transgene products after transplantation of GFP-transduced hematopoietic stem cells.

Tacrolimus binds to FKBP, and cyclosporine binds to cyclophilin. Both complexes exert their activity by inhibiting the calcineurin, a phosphatase that facilitates NFAT translocation to the nucleus resulting in the upregulation of IL-2. As such, blocking IL-2 activity results in a loss of T-cell activity, further elucidating the role of T-cell immunity in the response against GFP [30, 31].

Other immunological interactions that have been shown to prolong the in vivo survival of GFP-labeled cells including the depletion of immune cells [32], use of conventional immunosuppressants [33], use of stem/progenitor cells’ as immune modulators [34] and limiting the amount of antigen, and immune system activation by the antigen-presenting cells [29].

There have been experiments exploring possible ways to minimize the immunological cytotoxicity in GFP experiments. It has been shown that C57BL/6 have low immunogenic properties compared to other mice strains such as BALB/c and *mdx* [6, 9]. Also, with in vivo studies, using mice myoblast cells, it has been demonstrated that GFP transgenic mice cells displayed more effective engraftment capabilities and lasted longer, compared to GFP-transduced cells, and therefore are better candidates for dynamic in vivo tracking [9].

Epitope prediction and binding affinity studies revealed that mouse MHC Class I (H2-Kd) molecules function as a naturally occurring epitope of eGFP, indicating T-cell activation cascade [10]. This finding discovered in an experiment indicating that effector lymphocytes, accountable for eliciting a response against foreign antigens, did not display a response to the transplantable sarcoma cells, but did display strong cytotoxicity against the eGFP-expressing sarcoma cells [10].

It has been demonstrated that transfection and transduction can be immunogenic in in vivo experiments [35, 36]. This effect should be differentiated from direct GFP immunogenicity. Unfortunately appropriate controls are not included in many of the papers describing GFP immunogenicity. An appropriate control would be transfection or transduction with an empty vector. One paper that includes this appropriate control is Liu et al. who showed that GFP-transfected cells, compared to the empty-vector transfected cells, have higher levels of CPP32, an indicator of apoptosis. However, both the groups demonstrated higher levels of CPP32 compared to non-transfected cells [13]. In another experiment comparing different analogs of GFP, hepatic cells transfected with GFP, failed to produce stable progenies compared to the cells transfected with GFP analogs [21]. This experiment shows that GFP has special toxicity, independent of the transfection itself.

## Cytotoxicity of GFP (Table 1)

GFP is cytotoxic by a variety of mechanisms in addition to immunogenicity.

Initiation of the apoptosis cascade has been postulated as a possible mechanism for the toxicity of GFP and cellular death. After being transfected by various GFP-plasmid vectors, mouse embryonic and baby hamster kidney fibroblast cells lost their GFP signals and disappeared after 120 h [13]. Subsequently, various morphological (loss of structural integrity) and molecular changes (redistribution of phosphatidylserine, an indicator of the apoptosis signaling cascade initiation, to the cell surface) consistent with GFP-induced apoptosis were reported in this experiment. Another valuable finding suggestive of cellular apoptosis was presence of CPP32 (Caspace-3, an apoptotic protein) after fading of GFP signals [13]. CPP32, a member of interleukin-1β converting enzyme (ICE) family, plays an important role in programed cell death. It has been shown that the CPP32 is highly expressed in cells initiating apoptosis. In contrast, inactivation of CPP32 dramatically reduces apoptosis; therefore, CPP32 activity measurement is a reliable tool for monitoring apoptosis [37, 38]. Consistent with morphological changes implying apoptosis, CPP32 expression level is increased in cells transfected with GFP-plasmid compared to empty-vector transfected cells [13].

In addition to initiating the apoptosis cascade, reactive oxygen production induced by GFP has been linked to cellular toxicity and eventual death in GFP expressing cells. It was previously described that MHC class I (H2-Kd) is the naturally occurring epitope of eGFP, initiating the activation of CTLs [10]. However, it remains unclear how the immunogenicity of GFP through the MHC I pathway is related to the elevated reactive oxygen species (ROS) observed in various experiments. One proposed mechanism is through the exocytosis of granzyme B (GrB) through activated CTLs. Activated CTLs induce the exocytosis of GrB, perforin and GrA through the death receptor, FAS/FASL. GrB, once exocytosed facilitates the release of mitochondrial ROS through the direct cleavage of caspace-3 and nuclear lamin [39, 40].

Enhanced sensitivity of GFP expressing cells to anticancer drugs, such as Etoposide, has been associated with increased levels of ROS in cells. This finding is confirmed by increased levels of p53-dependent glutathione, which acts as a cellular defense mechanism in oxidative stress situations [41, 42]. Owing to oxidative stress, neuroblastoma cell lines, which lack CD80, showed increased sensitivity to cytotoxic agents when transduced with GFP, eGFP, and YFP [42]. CD80 is a protein on the activated B-cells and is necessary for co-stimulation signal required for activation of T-cells. It was demonstrated that, independent from immunogenicity, both CD80-negative and CD80-transduced neuroblastoma cells had significantly enhanced sensitivity to cytotoxic anticancer agents when transduced with GFP, eGFP, and YFP, [42].

The cellular toxicity of GFP and the cellular toxicity of GFP analogs are not the same. Cells transfected with GFP are less stable than those transfected with analogous fluorescent proteins, such as CFP, YFP [21]. While investigating rat liver cells [22, 43], it was found that the GFP-transfected liver cells yielded 50-fold fewer colonies when compared to the same cell type transfected with CFP or YFP gene constructs. Moreover, colonies that were transfected with GFP were unable to propagate as stable cell strains, where colonies transfected with CFP and YFP reached 100 % cloning efficiency [21].

Normally, an intact cell membrane is impermeable to propidium iodide, a molecule that can be used to stain cells by attaching to nucleic acids. Liver cells transfected with GFP plasmid have been shown to be permeable to propidium iodide. This could be a result of increased cellular permeability following the initiation of cellular death. The entry of propidium iodide into the cell leads to exhibition of red fluorescence after attachment to cellular DNA and RNA, which can be distinguished, from GFP signal. Transfection of rat liver cells with different GFP-plasmids resulted in similar consequences [21].

In addition to general GFP cellular toxicity, there has been some evidence that imply organ specific GFP cytotoxicity. GFP overexpression in the heart caused wild-type mice strains to develop dilated cardiomyopathy [44]. In this model, GFP expression was associated with significant increase in heart-to-body weight ratio, four-chamber dilation and thin myocardium suggestive of dilated cardiomyopathy in young rats. Observation of more severe cardiomyopathy in cells with higher GFP expression suggests dose-dependent effects [44].

Impairment of the actin-myosin interaction has also been reported due to GFP cytotoxicity. eGFP-transduction of myotubes using a lentivirus vector demonstrated impaired excitation-contraction coupling and diminished contractile function of myotubes due to GFP binding to the myosin-actin binding site [45, 46]. Surprisingly enough, [47], reactive gliosis and apoptosis of the forebrain area, due to neurodegeneration and cellular death, was evidenced in co-expression of eGFP and β -galactosidase.

In order to detect GFP in living cells, researchers must utilize light/laser or photoactivation, which uses precise light wavelengths, to excite the GFP. Photoactivation have been found to induce phototoxic effects [24, 48–51]. As a consequence, GFP’s cytotoxic effects can be complemented with the phototoxic effects of wavelength and light intensity during GFP excitation [52].

## Strategies for Utilizing GFP

GFP has been widely utilized as a gene expression marker for tracking cell progeny in animal models in translational research experiments. However, GFP may result in variable outcomes as a cell marker because GFP-expressing cells are liable to death from immunogenicity, free radical oxygenation, apoptosis, and other mechanisms. This is particularly important to consider when reviewing recent studies where GFP has been used to label or trace cells in a wide range of experiments including, but not limited to, dermal component cells [53], bone marrow derived stem cells/progenitor cells [54], hepatocytes [7], cardiomyocytes and neurons [55, 56]. Loss of GFP-labeled cells results in underestimation of the progeny of the original cohort.

The appropriate use of GFP is key to gathering reliable data in animal experiments that require cell tracking. For instance, a study using eGFP plasmid with a CMV promoter to track the propagation of prostate cells was deemed insignificant due to the cytotoxicity of eGFP [57]. One way to assess the cellular damage caused by eGFP is the utilization of different promoters. For example, eGFP under the control of a CMV promoter and d2eGFP under the control of a damage-inducible promoter can be used in conjunction to assess cytotoxicity. The CMV promoter allows eGFP to be continuously expressed in the cell. The d2eGFP protein contains an amino acid sequence at its C-terminus, which tags it for degradation, giving it a shorter half-life. This variant of GFP would only be expressed if particular transcription factors related to cell damage were expressed [58]. The cellular expression level of the d2eGFP could estimate the eGFP-induced cellular damage [58].

Another possible practical method to minimize GFP toxicity is to choose appropriate animal models. For instance, where applicable, C57BL/6 should be favored over other mice strains for cell tracking due to the minimal immunogenicity to GFP [11]. In contrast to C57BL/6 mice, heightened immunogenic response to GFP is seen following Respiratory Syncytial Virus (RSV) infection in BALB/c mice. They express significantly higher levels of IFN-γ, MCP-1, IL-6, and TNF-α. This is due to immunodominant H-2^d^ CTL in BALB/c mice, resulting in prolonged cytokine secretion and CTL response [59].

Additionally, cells obtained from GFP transgenic mice should be preferred over GFP-transduced cells due to their longevity. It is interesting that GFP immunogenicity and cytotoxicity has been demonstrated in multiple animal models. For example immunogenicity of GFP has been explored in mouse, rat, dog and monkey models while the possible toxic effect of GFP is investigated in mice and baby hamster. Many research experiments are done on large animals such as pigs, although they share almost a similar immune system with other animal models, no data is available regarding GFP immunogenicity and cytotoxicity in pigs.

Alternatively, GFP can be avoided altogether by using a different reporter gene based on the type of cell being studied. For cell lines that are sensitive to oxidative stress and immune response, GFP analogs should be considered in comparison to GFP. Analogs such CFP and YFP may be less cytotoxic expression markers for tracking cells and should be further explored [10, 13, 21–23].

## Conclusion

The problem in the use of GFP for cell tracking is that when a cohort of cells is labeled with GFP; their failure to track to an expected destination can be explained by GFP related cell toxicity rather than by basic biological mechanism. This means that negative tracing experiments cannot be reliably interpreted without careful consideration of GFP toxic effects.

## Abbreviations

GFP: Green fluorescent Protein
eGFP: Enhanced green fluorescent protein
CFP: Cyan fluorescent protein
YFP: Yellow fluorescent protein
RFP: Red fluorescent protein
H2-Kd: Mouse MHC class I.
IFN gamma: Cytokine that is critical for innate and adaptive immunity against viral, some bacterial and protozoal infections
MHC: Major histocompatibility complex
P53: The tumour suppressor p53 induces cell death by launching several pathways that are either dependent on or independent of gene transcription
ROS: Reactive oxygen species
TNF-α: Tumor necrosis factor α
NFAT: Nuclear factor of activated T-cells
CPP32: Caspace-3
